# SGLT2 inhibitor therapy and lower incidence of iron deficiency anaemia in patients with type 2 diabetes: A retrospective cohort study from Germany

**DOI:** 10.1111/dom.70057

**Published:** 2025-08-22

**Authors:** Theresia Sarabhai, Karel Kostev

**Affiliations:** ^1^ Department of Endocrinology, Diabetes and Metabolism University Hospital Essen, University Duisburg‐Essen Essen Germany; ^2^ Epidemiology IQVIA Frankfurt am Main Germany; ^3^ University Clinic Philipps, University Marburg Germany

**Keywords:** iron deficiency anaemia, real‐world evidence, retrospective cohort study, SGLT2 inhibitors, type 2 diabetes mellituserythropoiesis

## Abstract

**Aims:**

Iron deficiency anaemia (IDA) is a common comorbidity in patients with type 2 diabetes mellitus (T2DM). Sodium–glucose cotransporter‐2 inhibitors (SGLT2i) have been shown to modulate erythropoiesis and iron metabolism, but their association remains unclear. This cohort study investigated whether SGLT2i use, compared to dipeptidyl peptidase‐4 inhibitors (DPP‐4i), is associated with a lower incidence of IDA in T2DM.

**Materials and methods:**

We analysed data from the IQVIA™ Disease Analyzer, a German electronic medical records database (ICD‐10) from office‐based practices. Patients with T2DM (≥18 years) who initiated SGLT2i or DPP‐4i therapy alongside metformin between 2012 and 2022 were included. Those with prior anaemia diagnoses were excluded. Propensity score matching (1:1) was performed based on age, sex, baseline HbA1c, duration of metformin use, and anaemia‐related comorbidities. Kaplan–Meier analyses and Cox proportional hazards regression models were used to compare IDA incidence between cohorts over a 5‐year follow‐up.

**Results:**

In total 28 441 propensity score matched patients per cohort were analysed. The cumulative 5‐year incidence of IDA was significantly lower in the SGLT2i (6.9%) versus DPP‐4i cohort (11.3%) (*p* < 0.001). SGLT2i use was associated with a decreased risk of IDA (HR: 0.67; 95% CI: 0.58–0.78). Subgroup analyses confirmed this association in males and elder patients (>60 years), while the protective effect was limited to metformin use under 3 years.

**Conclusion:**

SGLT2i therapy was associated with a lower incidence of IDA in T2DM patients compared to DPP‐4i therapy. These findings suggest a potential hematologic benefit of SGLT2 inhibitors, warranting further investigation in randomised controlled trials.

## 1 INTRODUCTION

Sodium–glucose cotransporter 2 (SGLT2) inhibitors have been shown to improve iron deficiency anaemia in patients with various chronic diseases, including heart failure, chronic kidney disease with and without type 2 diabetes mellitus (T2DM).^1^, ^2^, ^3^ These effects extend beyond their glucose‐lowering capacity and suggest that SGLT2 inhibitors modulate iron metabolism and erythropoiesis through mechanisms not shared by other antidiabetic agents.^4^, ^5^, ^6^ Thus, the interaction between SGLT2 inhibitors and iron metabolism in type 2 diabetes remains an evolving area of interest.

Anaemia is a frequent comorbidity in T2DM, contributing to fatigue, cardiovascular risk, and the progression of diabetic complications.^7^ Besides anaemia of chronic disease and B12 deficiency, iron deficiency anaemia (IDA) is the most prevalent form in people with T2DM.^8^ While multifactorial in origin, IDA in T2DM is often related to chronic inflammation, functional iron deficiency, and impaired erythropoietin signalling—particularly in patients with diabetic nephropathy.^4^ IDA has also been associated with poor glycaemic control, reduced quality of life, and increased hospitalization risk in people with diabetes.^7^ Emerging evidence also implicates iron dysregulation and ferroptosis—a form of regulated, iron‐dependent cell death—as key contributors to β‐cell dysfunction, insulin resistance, and the progression of diabetes‐related organ damage.^9^, ^10^ Thus, ferroptosis also plays a crucial role in the pathogenesis of T2DM.^4^ Additionally, iron overload in liver and adipose tissues disrupts mitochondrial function and promotes insulin resistance, while altered iron signalling may also affect circadian rhythms and the gut‐brain axis in diabetes.^11^, ^12^ These findings underscore the importance of maintaining iron homeostasis for metabolic regulation.

SGLT2 inhibitors widely used for their cardio‐renal and glycaemic benefits in T2DM may influence iron homeostasis by stimulating erythropoiesis and increasing iron utilization. SGLT2 inhibitors have consistently been associated with favourable changes in iron‐related biomarkers, including reductions in ferritin and hepcidin and increases in serum transferrin receptor protein.^13^, ^14^ In contrast, dipeptidyl peptidase‐4 (DPP‐4) inhibitors—another widely used glucose‐lowering drug class—have shown no significant effects on iron metabolism or erythropoiesis.^15^ This makes DPP‐4 inhibitors an ideal comparator in observational studies to help isolate SGLT2‐specific effects and reduce confounding by indication.

To our knowledge, this is the first large‐scale, propensity score‐matched cohort study to evaluate the association between SGLT2 inhibitor use and incident clinically diagnosed IDA in a homogeneous population of patients with T2DM, using real‐world data from a German nationwide primary care database.

In this retrospective propensity score–matched cohort study, we examined whether treatment with SGLT2 inhibitors is associated with a lower incidence of clinically diagnosed iron deficiency anaemia in patients with T2DM compared to DPP‐4 inhibitors over a 5‐year observation period.

## 2 MATERIALS AND METHODS

### 2.1 Data source

This analysis was based on data from the IQVIA™ Disease Analyzer database, which contains case‐based information provided by approximately 3500 office‐based physicians (both GPs and specialists) in Germany. Information is available on patient demographics, drug prescriptions, and diagnoses. The Disease Analyzer database contains data from more than eight million patients, captured between 2012 and 2022. The sample of practices included is geographically representative for Germany, covering eight major German regions. In Germany, the sampling methods used for the selection of physicians' practices are appropriate for obtaining a representative database of general and specialised practices.^16^

### 2.2 Study population and covariables

**FIGURE 1:**
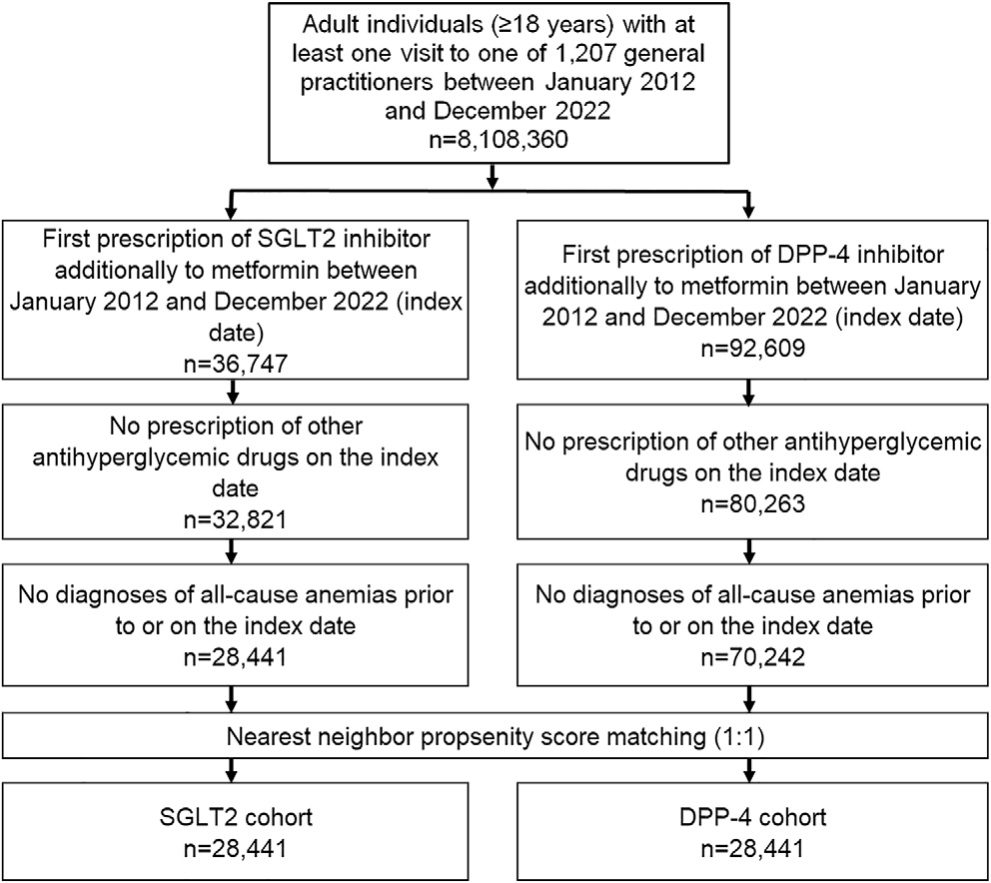
Selection of the study population: matching and cohort stratification process.

This retrospective cohort study includes patients aged ≥18 years with a diagnosis of type 2 diabetes (ICD‐10: E11) and a prescription of either SGLT‐2 inhibitor (ATC: A10BK) or DPP‐4 inhibitor (ATC: A10BH) additionally to metformin (ATC: A10BA) between January 2012 and December 2022. Patients were assigned to the SGLT2 or DPP‐4 cohort based on the earliest prescription date of either drug during the study period. Those who received both medications on the same index date, had prescriptions for other glucose‐lowering drugs at baseline, or switched between SGLT2 and DPP‐4 therapy during follow‐up were excluded. Furthermore, patients with diagnoses of all‐cause anaemias (ICD‐10: D50‐D64) prior to or on the index date were excluded. Participants who died during follow‐up were censored at the date of death. Patients were categorized into one of two cohorts: the SGLT2 cohort and DPP‐4 cohort; both cohorts were then compared with each other (Figure 1).

The covariables used in this study included age, sex, year of therapy begin (between 2012 and 2022), HbA1c value at baseline, and diagnoses which have been reported to be associated with anaemia, including gastrointestinal cancers, further gastrointestinal diseases (i.e., Crohn's disease, ulcerative colitis, gastrointestinal ulcers, colorectal polyps, gastrointestinal haemorrhage) documented within 12 months prior to or on index date; fractures and injuries documented within 3 months prior to or on index date; aspirin prescriptions documented within 3 months prior to or on index date; and duration of metformin use prior to index date.

### 2.3 Study outcome and statistical analyses

The study outcome was the cumulative incidence of iron deficiency anaemia (ICD‐10: D50) in patients treated with SGLT2 and DPP‐4. Patients were followed up to 5 years from the index date until the first iron deficiency anaemia diagnosis or change of antihyperglycaemic therapy (i.e., another drug instead or additionally to study therapy), or end of loss‐to‐follow‐up, or end of the database (31 December 2023), whichever occurred first.

Nearest neighbour propensity score matching (1:1) of SGT2 patients to DPP‐4 patients was conducted based on age, sex, HbA1c value, year of therapy begin, co‐morbidities previously listed, aspirin therapy, and metformin therapy duration. Standardized mean difference (SMD) of less than 0.1 was allowed, indicating that adequate covariate balance has been achieved.

The Kaplan–Meier method was used to estimate the differences between the SGLT2 and DPP‐4 cohorts in the percentages of patients with iron deficiency anaemia diagnosis within up to 5 years following the therapy initiation.

An univariable conditional Cox regression analysis was then used to investigate the association between SGLT2 therapy compared to DPP‐4 therapy and iron deficiency anaemia in matched cohorts stratified by age group, sex, and duration of metformin use (<1 year, 1–3 years, >3 years). The stratified analysis by duration of metformin therapy aimed to explore the potential impact of metformin‐associated B12 deficiency on the observed outcome. As propensity score matching was performed on key baseline variables—including age, sex, HbA1c, anaemia‐related comorbidities, aspirin use, and metformin duration—additional multivariable adjustment was not applied to avoid model overfitting and preserve the matched design.

Finally, a sensitivity analysis was performed using multivariable Cox regression, adjusting for prescriptions of proton pump inhibitors (PPIs), H2‐receptor antagonists, and non‐steroidal anti‐inflammatory drugs (NSAIDs) within 6 months prior to or on the index date. The results of the Cox regression analyses were presented as hazard ratios (HR) with corresponding p‐values, where *p*‐values below 0.01 were considered statistically significant due to multiple comparisons. All statistical computations were conducted using SAS version 9.4 (SAS Institute, Cary, NC, USA).

## 3 RESULTS

### 3.1 Baseline characteristics of study patients

After 1:1 propensity score matching, a total of 28 441 patients were included in each cohort, with 28 441 patients treated with SGLT2 inhibitors and 28 441 patients treated with DPP‐4 inhibitors available for analysis (Figure 1). Table 1 presents the baseline characteristics of the study population. The mean age was 64.3 years in the SGLT2 cohort and 64.9 years in the DPP‐4 cohort. The proportion of female patients was 36.5% in the SGLT2 group and 40.2% in the DPP‐4 group. Regarding prior metformin use, most patients had <1 year of metformin exposure (SGLT2: 64.3%; DPP‐4: 66.4%), followed by 1–3 years and >3 years of use, with a comparable distribution between groups. HbA1c levels were comparable between groups, with a mean of 8.0% in both cohorts. The prevalence of chronic kidney disease (17.2% vs. 16.2%), colorectal disease (5.9% vs. 5.2%), gastrointestinal cancers (3.0% vs. 2.7%), and recent injuries or fractures (19.5% vs. 19.6%) was similar in both groups. Prescriptions for aspirin within 3 months prior to or on the index date were slightly more common in the SGLT2 cohort (18.5% vs. 15.2%).

**TABLE 1:** Baseline characteristics of the 1:1 propensity score matched study cohorts: SGLT2 inhibitor versus DPP‐4 inhibitor users.

Variable	SGLT2 users	DPP‐4 users	SMD
*N*	28 441	28 441	
Age (mean, SD)	64.3 (12.7)	64.9 (13.0)	−0.049
≤50 years (%)	3859 (13.6)	3758 (13.2)	
51–60 years (%)	6929 (24.4)	6696 (23.5)	
61–70 years (%)	8387 (29.5)	8037 (28.3)	
>70 years (%)	9266 (32.5)	9950 (35.0)	
Sex: female (%)	10 378 (36.5)	11 441 (40.2)	−0.038
Sex: male (%)	18 063 (63.5)	17 000 (59.8)	
Year of therapy begin: 2012–2015	1175 (4.1)	1175 (4.1)	0.001
Year of therapy begin: 2016–2019	8874 (31.2)	8911 (31.3)	
Year of therapy begin: 2020–2022	18 392 (64.7)	18 355 (64.6)	
Metformin therapy duration <1 year	18 298 (64.3)	18 887 (66.4)	0.035
Metformin therapy duration 1–3 years	4093 (14.4)	3913 (13.8)	
Metformin duration >3 years	6050 (21.3)	5641 (19.8)	
Injuries and fractures within 3 months prior to or on index date	5554 (19.5)	5579 (19.6)	0.000
Colorectal diseases	1673 (5.9)	1474 (5.2)	−0.003
Gastrointestinal cancers	841 (3.0)	765 (2.7)	−0.007
Chronic kidney disease	4904 (17.2)	4605 (16.2)	−0.011
Aspirin prescriptions within 3 months prior to or on index date	5274 (18.5)	4334 (15.2)	−0.033
HbA1c (%) (mean, SD)	8.0 (1.7)	8.0 (1.7)	0.000

Abbreviations: DPP‐4, dipeptidyl peptidase‐4; SGLT‐2, sodium‐glucose cotransporter‐2.

### 3.2 Incidence of iron deficiency anaemia and Kaplan–Meier analysis

**FIGURE 2:**
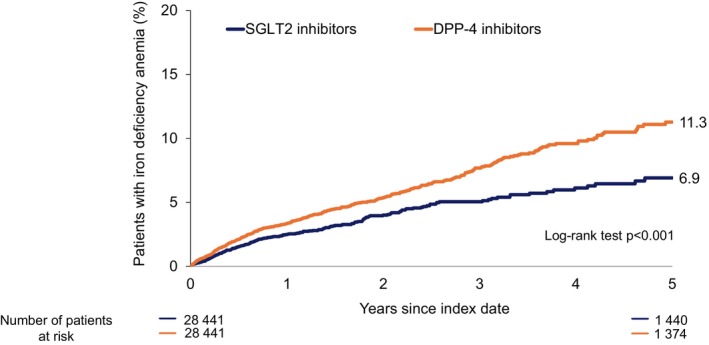
Cumulative incidence and number at risk of iron deficiency anaemia over 5 years in patients with type 2 diabetes treated with SGLT2 versus DPP‐4 inhibitors. DPP‐4, dipeptidyl peptidase‐4; SGLT‐2, sodium‐glucose cotransporter‐2.

Over a 5‐year follow‐up period, patients treated with SGLT2 inhibitors exhibited a significantly lower cumulative incidence of iron deficiency anaemia compared to those receiving DPP‐4 inhibitors (*p* < 0.001) (Figure 2). The 5‐year cumulative incidence of iron deficiency anaemia was 11.3% in the DPP‐4 cohort and 6.9% in the SGLT2 cohort. The Kaplan–Meier curves revealed an early and progressive divergence in event rates between the two groups, beginning within the first year of follow‐up and continuing throughout the study period, suggesting a sustained protective effect of SGLT2 inhibitor therapy over time.

### 3.3 Association between SGLT2 therapy and the risk of iron deficiency anaemia

The Cox regression analysis (Table 2) indicated that SGLT2 inhibitor use was associated with a significantly lower incidence of iron deficiency anaemia in the total study population (HR: 0.67; 95% CI: 0.58–0.78; *p* < 0.001). In age‐stratified analyses, the association was significant in the age groups 61–70 years (HR: 0.68; 95% CI: 0.51–0.91; *p* = 0.009) and >70 years (HR: 0.73; 95% CI: 0.59–0.91; *p* = 0.006). SGLT2 inhibitor use was associated with HR below 1.0 across most subgroups, including patients aged ≤50 years (HR: 0.74; 95% CI: 0.47–1.19) and 51–60 years (HR: 0.71; 95% CI: 0.48–1.05) (Table 2). This association remained significant in both female (HR: 0.73; 95% CI: 0.58–0.91; *p* = 0.005) and male patients (HR: 0.64; 95% CI: 0.52–0.79; *p* < 0.001). Sex‐ and age‐stratified models revealed heterogeneity in the strength of association between the sexes. Among female patients aged ≤50 years, the point estimate suggested a reduced risk of IDA with SGLT2 inhibitor use (HR: 0.56; 95% CI: 0.32–0.99; *p* = 0.044). No associations were observed in older female subgroups. In contrast, male patients demonstrated associations in the 61–70 age group (HR: 0.56; 95% CI: 0.38–0.83; *p* = 0.004), while findings in the 51–60 (HR: 0.55; 95% CI: 0.32–0.94; *p* = 0.028) and >70 years group (HR: 0.73; 95% CI: 0.55–0.98; *p* = 0.034) demonstrated a trend towards an association. Overall, while the direction of effect generally favoured SGLT2 inhibitor use across most subgroups, significant association was more robust in older male patients. A significant association between SGLT2 inhibitor use and lower IDA incidence was observed in patients with metformin exposure of <1 year (HR: 0.72; 95% CI: 0.59–0.88; *p* = 0.002) and <3 years (HR: 0.73; 95% CI: 0.61–0.88; *p* < 0.001). No association was found among patients with 1–3 years (HR: 0.78; 95% CI: 0.52–1.16; *p* = 0.218) or >3 years (HR: 0.86; 95% CI: 0.64–1.16; *p* = 0.320) of metformin use, suggesting that the protective association may be confined to patients with shorter metformin exposure. Also, after adjustment for PPIs, H2‐receptor antagonists, and NSAIDs, SGLT2 inhibitors remained significantly associated with a reduced risk of anaemia (HR 0.70; 95% CI 0.60–0.81). The overall absolute risk reduction was 4.4 percentage points, corresponding to a number needed to treat of approximately 23 to prevent one case of iron deficiency anaemia over 5 years (Figure 2).

**TABLE 2:** Absolute numbers at risk, events and associations between incidence of iron deficiency anaemia and SGLT2 versus DPP‐4 inhibitors.

Cohorts	Number of patients at risk in the SGLT2 cohort	Number of patients at risk in the DPP‐4 cohort	Number of events in the SGLT2 cohort	Number of events in the DPP‐4 cohort	Hazard ratio (95% CI)	*p* value
Total	28 441	28 441	254	491	0.67 (0.58–0.78)	<0.001
Patients aged ≤50 years	3859	3758	29	44	0.74 (0.47–1.19)	0.214
Patients aged 51–60 years	6929	6696	39	69	0.71 (0.48–1.05)	0.086
Patients aged 61–70 years	8387	8037	70	132	0.68 (0.51–0.91)	0.009
Patients aged >70 years	9266	9950	116	246	0.73 (0.59–0.91)	0.006
Female patients	10 378	11 441	118	217	0.73 (0.58–0.91)	0.005
Female patients aged ≤50 years	1272	1265	18	35	0.56 (0.32–0.99)	0.044
Female patients aged 51–60 years	2313	2241	20	24	0.98 (0.55–1.76)	0.949
Female patients aged 61–70 years	3167	2941	34	46	0.92 (0.60–1.42)	0.705
Female patients aged >70 years	4667	3935	46	112	0.72 (0.51–1.02)	0.065
Male patients	18 063	17 000	136	274	0.64 (0.52–0.79)	<0.001
Male patients aged ≤50 years	2455	2602	11	9	1.57 (0.63–3.90)	0.333
Male patients aged 51–60 years	4336	4657	19	45	0.55 (0.32–0.94)	0.028
Male patients aged 61–70 years	4922	5436	36	86	0.56 (0.38–0.83)	0.004
Male patients aged >70 years	5301	5356	70	134	0.73 (0.55–0.98)	0.034
Metformin use <3 years	22 391	22 800	183	371	0.73 (0.61–0.88)	<0.001
Metformin use <1 year	18 298	18 887	146	301	0.72 (0.59–0.88)	0.002
Metformin use 1–3 years	4093	3913	37	70	0.78 (0.52–1.16)	0.218
Metformin use >3 years	6050	5641	71	120	0.86 (0.64–1.16)	0.320

*Note*: Age‐, sex‐, and metformin use duration‐stratified cox regression analysis of iron deficiency anaemia in SGLT2 users versus DPP‐4 inhibitor users.

Abbreviations: DPP‐4, dipeptidyl peptidase‐4; SGLT‐2, sodium‐glucose cotransporter‐2.

## 4 DISCUSSION

This retrospective propensity score‐matched cohort study with a 5‐year follow‐up period analysed real‐world data to assess the incidence of iron deficiency anaemia associated with SGLT2 inhibitor use among 28 441 patients with T2DM treated in German primary care practices. Over a 5‐year observation period, our findings revealed a significant association between SGLT2 inhibitor therapy and a lower incidence of iron deficiency anaemia compared to DPP‐4 inhibitor therapy. The analysis employed rigorous propensity score matching to balance baseline characteristics and minimize confounding, comparing outcomes between patients initiating treatment with SGLT2 inhibitors versus those treated with DPP‐4 inhibitors. Patients using SGLT2 inhibitors had a significantly reduced risk of IDA compared to those receiving DPP‐4 inhibitors, with consistent results observed particularly in male patients and in older age groups (>60 years). In contrast, only a trend towards an association was observed among female and younger patients. Stratified analysis by metformin exposure further indicated that the protective association was limited to individuals with shorter metformin use (<1 and <3 years), whereas no effect was observed in those with longer‐term exposure (>3 years). These data underscore the potential benefits of SGLT2 inhibitors beyond glycaemic control, suggesting a favourable effect on iron homeostasis, especially in selected patient subgroups.

Our findings align well with previous studies demonstrating hematologic benefits associated with SGLT2 inhibitor use. For example, the EMPEROR‐Reduced trial (empagliflozin), which primarily focused on patients with heart failure with reduced ejection fraction regardless of diabetes status, demonstrated improvements in haemoglobin and haematocrit levels by about 2% over 52 weeks.^17^ Similarly, the DAPA‐HF trial (dapagliflozin), involving heart failure patients with or without diabetes, observed significant increases in haematocrit and reductions in anaemia incidence following dapagliflozin treatment.^18^ Comparable effects have been shown for canagliflozin in people with chronic kidney disease and type 2 diabetes over a 2‐year period.^19^ Initiation of SGLT2 inhibitors was accompanied by an increase in haemoglobin, which averages ≈0.7 g/dL, with a mean achieved haemoglobin level of ≈14.5 g/dL; the magnitude of the increase is similar in patients with and without anaemia, indicative of better iron utilisation and erythropoiesis.^17^, ^18^ These increases were apparent after 4–12 weeks and were sustained for 1–3 years of follow‐up.^20^ Anaemia was more than twice as likely to be corrected in patients receiving SGLT2 inhibitors as compared with placebo over a 2‐year therapy.^17^, ^18^ Approximately 50%–70% of treated patients achieved haemoglobin and haematocrit levels in the nonanaemic range. This correction of anaemia by SGLT2 inhibition was observed although <10% of patients received iron supplements or other treatments for anaemia.^20^ These previous results are in agreement with our study, which demonstrated an early and sustained reduction in IDA incidence among SGLT2 users compared to DPP‐4 users.

While these previous studies provide robust evidence of SGLT2 inhibitors' hematologic effects, our study extends these findings to a homogenous population of patients with T2DM without interfering chronic disease and over a longer observation period (5 years). We isolate IDA in people with T2DM and confirm the positive impact of SGLT2 inhibitors in this specific clinical setting. Notably, we observed a threefold lower rate of IDA with SGLT2 use compared to DPP‐4 use, likely due to the more homogenous selection of a cohort with low prevalence of chronic kidney disease. The lower incidence rate of IDA in the SGLT2 inhibitor cohort might correspond with recent mechanistic insights. SGLT2 inhibitors have been shown to modulate iron metabolism through several mechanisms. Preclinical and clinical studies suggest that these agents reduce serum hepcidin levels—a key negative regulator of iron absorption and mobilization—thereby facilitating greater iron availability for erythropoiesis.^21^, ^22^ In addition, SGLT2i may decrease circulating ferritin, an acute‐phase reactant, further indicating a reduction in chronic low‐grade inflammation. Improved erythropoiesis may also result from enhanced erythropoietin production triggered by mild plasma volume contraction and increased renal hypoxia signalling following SGLT2 inhibition. These effects contribute to improved iron utilization and red blood cell production, which may explain the early and sustained rise in haemoglobin levels observed in clinical trials. These observations support our epidemiological findings, suggesting a biological plausibility for the protective effect of SGLT2 inhibitors against IDA.^22^

Importantly, we also observed that the protective association between SGLT2 inhibitors and reduced IDA incidence was restricted to patients with shorter metformin exposure (<1 year and <3 years), while no effect was found in long‐term metformin users (>3 years). This observation may reflect the influence of residual confounding, possibly related to metformin‐associated vitamin B12 deficiency, although this was not directly measured in our study.^23^ Given the distinct pathophysiological mechanisms of B12 deficiency anaemia (macrocytic) and iron deficiency anaemia (microcytic), this association should be interpreted with caution. The reduced effect in long‐term metformin users (>3 years) may be due to diagnostic misclassification or overlapping anaemia etiologies rather than a true loss of SGLT2i efficacy. Although both conditions can contribute to overall anaemia burden, further studies including laboratory‐confirmed anaemia subtypes are needed to better understand these interactions.

Further, IDA is common among older adults (≥60 years) and is associated with adverse outcomes, like frailty and reduced quality of life.^24^, ^25^ Differentiating iron deficiency from other causes of anaemia is essential for appropriate management, often involving oral or intravenous iron supplementation.^25^ IDA in men and postmenopausal women is most often due to occult gastrointestinal blood loss.^25^ In individuals with diabetes mellitus, this risk is further amplified by chronic hyperglycaemia, which contributes to altered iron metabolism and increased susceptibility to iron deficiency compared with normoglycaemic individuals.^26^ Between 2012 and 2022, anaemia prevalence among diabetic patients rose markedly from 15.3% to 40.7%,^8^ reflecting ageing, longer diabetes duration, increased comorbidities, and broader screening and medication use.^27^ The fact that the observed effects of reduced IDA incidence in SGLT2 users are particularly strong in the older age groups (≥60 years), particularly in men and less in women, is noteworthy. This finding may reflect the higher baseline prevalence of iron deficiency in the elderly due to cumulative comorbidities, age‐related changes in iron metabolism, and reduced dietary intake or absorption. It also suggests that SGLT2 inhibitors may exert their haematologic benefits more effectively in subgroups at higher baseline risk of iron deficiency. Further basic studies would be needed to understand this age‐associated phenomenon in more detail.

It has been demonstrated that women are more frequently affected by IDA than men, primarily due to menstruation, pregnancy, and nutritional deficiencies.^28^ Postmenopausal women have an increased risk of developing gastrointestinal bleeding and malabsorption syndromes. In contrast, the prevalence of IDA in men tends to increase with age, especially after the age of 65 years.^28^ In our subgroup analyses, the protective association of SGLT2 inhibitors was pronounced in older male patients but not in women after stratification by age. This observed sex difference may reflect underlying physiological and hormonal differences in iron handling and erythropoiesis. One potential mechanism is the role of testosterone, which has been shown to stimulate erythropoietin production and promote red blood cell formation, possibly enhancing the hematologic effects of SGLT2 inhibitors in men.^29^ In older individuals, the greater benefit may also be related to the anti‐inflammatory effects of SGLT2 inhibitors,^30^ which can lower hepcidin levels and improve iron mobilization in the context of chronic low‐grade inflammation common in aging. The potential for more pronounced effects at lower hematologic parameters merits further exploration through basic research.

A key methodological strength of our study is the direct comparison of SGLT2 inhibitors with DPP‐4 inhibitors. Previous large trials such as CREDENCE and DECLARE‐TIMI 58 compared SGLT2 inhibitors to placebo in heterogeneous populations.^31^, ^32^ In contrast, we selected DPP‐4 inhibitors as an active comparator due to their neutrality on erythropoiesis and iron metabolism.^15^ This active comparison minimises confounding by indication and supports the causal inference that observed IDA reductions are linked to SGLT2 therapy. The consistent effect in selected subgroups and early divergence of Kaplan–Meier curves further supports this relationship.

Nevertheless, several limitations must be acknowledged. First, the observational design precludes causal inference, and despite rigorous propensity score matching, residual confounding from unmeasured variables—such as ethnicity or race, deprivation status, physical activity, smoking, alcohol use, or diet—cannot be excluded. Second, socioeconomic data (e.g., education, income, occupation) were not available, though these factors may influence both anaemia risk and diabetes management. Third, IDA diagnoses relied on clinical coding without laboratory confirmation (e.g., ferritin, transferrin saturation, or hepcidin), potentially leading to underdiagnosis or misclassification. Fourth, data on out‐of‐counter use of iron, vitamin B12, or folate therapy were unavailable, limiting adjustment for confounding treatments. Lastly, genetic predispositions affecting iron metabolism and sex‐specific factors such as menopausal status were not accounted for. Despite these limitations, the large, well‐matched sample and subgroup results enhance the external validity and robustness of our findings. SGLT2 inhibitor therapy is associated with a reduced incidence of clinically diagnosed IDA in patients with T2DM, particularly in older adults and male patients. The association remained significant in those with shorter metformin exposure (<1 and <3 years), whereas no effect was observed among long‐term metformin users (>3 years), likely due to competing risks such as metformin‐induced vitamin B12 deficiency. These findings support the hypothesis that these agents improve iron handling and erythropoiesis via mechanisms beyond glucose control, although their effectiveness may vary depending on individual patient characteristics, including age, sex, and treatment history. Future prospective studies and mechanistic trials should clarify the underlying biological pathways and assess whether SGLT2 inhibitors can improve outcomes in people with established anaemia or iron metabolism disorders.^22^ Moreover, our real‐world, large‐scale T2DM cohort complements randomized controlled trial evidence by offering insights into routine clinical care, where comorbidities, medication use, and patient profiles often differ from those in controlled settings. The extended five‐year follow‐up strengthens our assessment of long‐term hematologic outcomes.

In conclusion, this study adds real‐world evidence for an association between SGLT2 inhibitor use and reduced incidence of IDA in people with T2DM, especially in older men and those with shorter metformin exposure. These findings supplement previously described haematological benefits of SGLT2 inhibitors and underscore their potential role in managing diabetes‐related anaemia in selected patient subgroups. Further studies are warranted to validate these findings and explore whether this hematologic benefit translates into improved clinical outcomes.

## AUTHOR CONTRIBUTIONS

T.S. and K.K. have designed, wrote and revised the manuscript. K.K. conducted data analysis and analysed data.

## FUNDING INFORMATION

This research did not receive any specific grant from funding agencies in the public, commercial, or not‐for‐profit sectors. T.S. work is supported by the DFG in the framework of the DFG Clinician Scientist Programme UMEA, FU 356/12‐2.

## CONFLICT OF INTEREST STATEMENT

The authors declare no conflict of interest.

## PEER REVIEW

The peer review history for this article is available at https://www.webofscience.com/api/gateway/wos/peer-review/10.1111/dom.70057.

## ETHICS STATEMENT

Ethical review and approval were waived for this study as studies that are strictly registry‐based do not require ethical approval or informed consent from participants, which was confirmed by the ethical committee of University Duisburg‐Essen. Patient data were analysed in aggregated form and individual data were not available.

## CONSENT

In accordance with national and European legislation, individual consent forms were not required or obtained for this study; this was confirmed by the ethical committee of University Duisburg‐Essen.

## Data Availability

The data that support the findings of this study are available from the authors TS and KK, upon reasonable request.
